# Veterinary Department, University of Pennsylvania

**Published:** 1901-08

**Authors:** S. J. J. Harger


### COMMENCEMENT EXERCISES.

### VETERINARY DEPARTMENT, UNIVERSITY OF PENNSYLVANIA.

THE commencement of the Veterinary Department of the University of Pennsylvania was held on June 12th, at 11 A.M., in the American Academy of Music. The exercises were participated in with the usual formality by all the departments of the University. The invocation was asked by Bishop Ozi Whitaker. The provost made a few introductory remarks. The orator of the day was Attorney-General James M. Beck, an alumnus of the institution, who made a brilliant address teeming with good advice to young men. After this the degrees were conferred. There were thirteen graduates: Harry E. Bender, Lititz, Pa.; Thomas S. Carlisle, Chestnut Hill, Pa.; Charles L. Colton, Englewood, N. J.; Samuel H. Gilleland, Marietta, Pa.; John W. Hayman, Pa.; Oscar M. Norton, Pa.; Reuben Rivers, North Wales, Pa.; Edward Seidel, Bryn Mawr, Pa.; Charles S. Shore, Hobblesville, Pa.; Fred. Steble, Jr., Falls of Schuylkill, Pa.; Charles R. Walter, Cunningham, Pa.; Frank J. Woodward, Lima, Pa.; Harry W. Watson, South Waterford, Maine.

Drs. Colton and Watson will remain as house surgeons of the hospital of the Veterinary Department; Dr. Shore will settle in Lake City, Minn.; Dr. Walter in Hazleton; Dr. Woodward at 5912 Market Street, Philadelphia, and the others will commence practice at their own towns.

The annual banquet was held in the evening at Soulas', and about fifty covers were laid. The following toasts were responded to: W. H. Ridge, "Truth;" E. M. Ranck, "Daring;" M. E. Conrad, "Accuracy;" J. Morris Carter, "Good Manners;" Howard Felton, "Diligence;" W. H. Hoskins, "Economy;" S. J. J. Harger, "Enthusiasm." These remarks brought forth wit as well as good advice to the young practitioner. Dr. C. J. Marshall, the President of the Alumni Association, was toastmaster. Letters of regret for absence were received from Drs. Leonard Pearson and R. S. Huidekoper.

Previous to the banquet a business meeting was held for the purpose of reorganizing the Association and infusing into it new life. A new Constitution and By-Laws were adopted, and means proposed to bring every alumnus in closer communion with his alma mater. The banquet was in every way a success.

S. J. J. HARGER.

